# Is preoperative hypoalbuminemia or hypoproteinemia a reliable marker for anastomotic leakage risk in patients undergoing elective colorectal surgery in an enhanced recovery after surgery (ERAS) program?

**DOI:** 10.1007/s00384-023-04450-5

**Published:** 2023-05-31

**Authors:** Joseph Do Woong Choi, Charlotte Kwik, Nurojan Vivekanandamoorthy, Aswin Shanmugalingam, Lachlan Allan, Fiona Gavegan, Karen Shedden, Ashleigh Peters, Toufic El Khoury, Nimalan Pathmanathan, James Wei Tatt Toh

**Affiliations:** 1 Department of Colorectal Surgery, Westmead Hospital, Corner Hawkesbury Road and Darcy Roads, Westmead, Sydney, NSW Australia; 2 Faculty of Medicine and Health, The University of Sydney, Sydney, NSW Australia; 3 School of Medicine, University of Notre Dame, Sydney, NSW Australia

**Keywords:** Anastomotic leak, Hypoalbuminemia, Hypoproteinemia, Immunonutrition

## Abstract

**Purpose:**

Preoperative hypoalbuminemia has traditionally been used as a marker of nutritional status and is considered a significant risk factor for anastomotic leak (AL).

**Methods:**

The Westmead Enhanced Recovery After Surgery (WERAS) prospectively collected database, consisting of 361 patients who underwent colorectal surgery with primary anastomosis, was interrogated. Preoperative serum albumin and protein levels (measured within 1 week of surgery) were plotted on receiver operating characteristic curves (ROC curves) and statistically analyzed for cutoff values, sensitivity, specificity, positive predictive values (PPV), and negative predictive values (NPV).

**Results:**

The incidence of AL was 4.4% (16/361). Overall mortality was 1.4% (5/361), 6.3% (1/16) in the AL group, and 1.2% (4/345) in the no AL group. The median preoperative albumin and protein level in the AL group were 39 g/L and 75 g/L, respectively. The median preoperative albumin and protein level in the no AL group were 38 g/L and 74 g/L, respectively. The Mann–Whitney *U* test showed no statistically significant difference in albumin levels (*p* = 0.4457) nor protein levels (*p* = 0.6245) in the AL and no AL groups. ROC curves demonstrated that preoperative albumin and protein levels were not good predictors of anastomotic leak. Cutoff values for albumin (38 g/L) and protein (75 g/L) both had poor PPV for AL (4.8% and 3.8% respectively).

**Conclusion:**

In patients undergoing elective colorectal surgery as part of an ERAS program, preoperative serum albumin and protein levels are not reliable in predicting AL. This may be because of nutritional supplementation provided as part of an ERAS program may correct nutritional deficits to protect against AL or that low albumin/protein is not as robust a marker of AL as previously reported.

## Introduction

Anastomotic leak (AL) leading to intra-abdominal sepsis remains a serious and unpredictable complication associated with restorative colorectal surgery. The reported incidence ranges from 2–7% when surgery is performed by experienced surgeons [1]. AL is associated with increased morbidity, mortality (12–30%), length of hospitalization, and hospital costs [2–4]. It often results in re-operation, and the need for a temporary or permanent stoma, which has a significant impact on the patients’ quality of life [5]. In addition, AL has been shown to be an independent risk factor of local recurrence, diminished overall, and cancer-specific survival after potentially curative colorectal cancer resection [6].

Adequate blood supply, tension-free tissue approximation as well as technical expertise may reduce the risk of AL but not necessarily negate it. Nutritional state has been traditionally considered to be an important factor contributing to AL. Although low serum albumin and protein are not necessarily a direct marker of nutritional status, low albumin and protein have often been used as markers of poor nutritional reserve in the surgical literature [7]. Several studies have reported an association between low serum albumin/protein levels and an increased incidence of AL [8–11] and that correction of malnutrition may reduce the risk of AL [12]. Additionally, several studies have also reported on the benefits of immunonutrition (containing key nutrients such as ω-3 fatty acids, arginine, and nucleotides). Immunonutrition is thought to reduce the postoperative production of proinflammatory lipid mediators and cytokines in addition to promoting lymphocyte production, function and tissue healing [13, 14].

While there has been increasing evidence that C-reactive protein is a useful marker to predict AL in colorectal surgery [15, 16], the association between low pre-operative albumin and protein with AL following colorectal surgery is based on a smaller number of studies. Thus, the primary aim of this study was to determine if preoperative serum albumin and protein may be used as a reliable biochemical marker in predicting AL among patients undergoing elective colorectal surgery as part of an ERAS program.

## Methods

### Study population

Patient data were derived from a single institution, with a retrospective review of a prospectively collected database—Westmead Enhanced Recovery After Surgery (WERAS). This consisted of 361 patients who underwent elective colorectal surgery with primary anastomosis (with or without defunctioning stoma) from January 2017 to December 2022. Emergency cases, minor procedures, palliative procedures, cases associated with bowel obstruction, and patients who did not have a primary anastomosis were excluded. All patients included in this study underwent stapled anastomosis (for colectomies, patients underwent stapled side-to-side anastomosis + / − suture reinforcement, for proctectomies, patient underwent end-to-side circular stapler anastomosis).

Preoperatively, all patients underwent ERAS counseling, and to improve their nutrition status and immunity, immunonutrition “Impact” (Novartis/Nestlé) was recommended three times a day for 1 week prior to surgery. Serum albumin and protein levels were taken approximately 1 week prior to surgery, prior to the commencement of immunonutrition. A day prior to surgery, patients were given 1 g neomycin and 400 mg metronidazole at 7 am, 3 pm, and 10 pm, with glycoprep (PEG) or picoprep (sodium phosphate) at 11 am, 2 pm, and 5 pm. Intraoperatively, prophylactic subcutaneous enoxaparin 40 mg or heparin 5000 IU was administered, and intravenous antibiotics were given at induction. The operation was performed either via an open, laparoscopic, laparoscopic converted to open, or hand-assisted laparoscopic (hybrid) technique using a gelport. Wound protectors, closing tray, and change of gloves were performed prior to closure. No abdominal drains were placed for right-sided colectomies. Postoperatively, patients had patient-controlled analgesia (PCA), regular metoclopramide, and ondansetron as required and were allowed to start free fluid diet. On postoperative day 1, indwelling urinary catheter was generally removed, and by postoperative day 2, patients were generally on a normal diet, with cessation of PCA.

### Data collection and ethical approval

A prospectively collected database with more than 50 data points was created on the Research Electronic Data Capture (REDCap) data management platform. The database was de-identified, with each patient assigned a patient identification (ID) number. Institutional ethics approval was granted, study reference number (5878) AU RED LNR/18/WMEAD/424. Data was collected prospectively by nurses, medical officers, and advanced trainees. Patients with morbidity, mortality, and prolonged stay were discussed at monthly ERAS meetings attended by consultant colorectal surgeon and team.

### Definition of AL

AL was defined as “a defect of the intestinal wall at the anastomotic site, leading to a communication between the intra and the extra-luminal compartments” as recommended by the International Study Group of Rectal Cancer [17]. Clinically, AL was suspected with increasing abdominal tenderness, peritonism, septicaemia, gas/fecal discharge from the abdominal drain, or organ failure. Routine computed tomography (CT) scan with hydro-soluble rectal contrast was not performed postoperatively unless AL was suspected. In this study, both clinical and/or radiological leaks were included as AL.

### Statistical analysis

Quantitative data are presented as median, inter-quartile ranges, and percentages. Differences between groups were evaluated using parametric or non-parametric tests. Qualitative, categorical data were analyzed using the chi-square test. Quantitative variables were analyzed using the Mann–Whitney *U* test to compare two groups. A receiver operating characteristic (ROC) curve was used to determine cutoff values, sensitivity, specificity, positive predictive values (PPV), and negative predictive values (NPV). The statistical analysis was performed using SPSS 23.0 package program (IBM SPSS Inc, USA). Statistical significance was set at a *p*-value of < 0.05.

## Results

During the study period, 361 patients underwent elective colorectal surgery with primary anastomosis. Emergency cases were excluded. The incidence of AL was 4.4% (16/361) with an overall mortality of 1.4% (5/361), 6.3% (1/16) in the AL group, and 1.2% (4/345) in the no AL group. AL was not influenced by gender (*p* = 0.837) nor body mass index (*p* = 0.5430) (Table 1).

**Table 1:** Patient demographics and clinical characteristics

**Characteristic**	**Total (*n* = 361)**	**No AL**	**AL**	***p* value**
Gender, *n* (%)				0.837
Male	167 (46.3%)	160 (46.4%)	7 (43.8%)	
Female	194 (53.7%)	185 (53.6%)	9 (56.2%)	
BMI (median, IQR)	27.7 (23.95–31.85)	27.8 (24–31.95)	27 (23.45–29.4)	0.543
Hospital stay (median, IQR)	6 (4–9)	6 (4–8)	20 (11.5–46.5)	< 0.00001
Procedure, *n* (%)				0.021
Right hemicolectomy	111 (31.0%)	111 (32.5%)	0 (0%)	
Transverse colectomy	4 (1.1%)	4 (1.2%)	0 (0%)	
Left hemicolectomy	16 (4.5%)	15 (4.4%)	1 (6.25%)	
Anterior resection (height not specified)	10 (2.8%)	10 (2.9%)	0 (0%)	
High anterior resection	95 (26.3%)	91 (26.7%)	4 (25%)	
Low anterior resection	47 (13%)	42 (12.3%)	5 (31.25%)	
Ultralow anterior resection	42 (11.6%)	37 (10.8%)	5 (31.25%)	
Total proctocolectomy and pouch with defunctioning ileostomy	16 (4.5%)	16 (4.7%)	0 (0%)	
Other restorative procedures	17 (4.8%)	16 (4.7%)	1 (6.25%)	
Approach				0.557
Open	35 (9.8%)	34 (10%)	1 (6.3%)	
Laparoscopic	239 (67.1%)	229 (67.4%)	10 (62.5%)	
Converted to open	32 (9%)	29 (8.5%)	3 (18.8%)	
Hand-assisted laparoscopic (hybrid)	50 (14%)	48 (14.1%)	2 (12.5%)	
Indication: colorectal cancer (CRC) vs non–CRC				1
CRC	285 (79%)	272 (78.8%)	13 (81.3%)	
Non-CRC	76 (21%)	73 (21.2%)	3 (18.7%)	
Crohn’s disease	6 (1.7%)	6 (1.7%)	0 (0%)	
Ulcerative colitis	3 (0.8%)	3 (0.9%)	0 (0%)	
Diverticulitis	33 (9.1%)	32 (9.3%)	1 (6.2%)	
Volvulus	1 (0.3%)	1 (0.3%)	0	
Others*	32 (8.9%)	30 (8.7%)	2 (12.5%)	
Pre-operative Albumin (median, IQR)	38 (36–41)	38 (36–41)	39 (34–39)	0.4457
Pre-operative Protein (median, IQR)	74 (71–79)	74 (71–79)	75 (73–78)	0.6245

*AL* anastomotic leak, *BMI* body mass index, *IQR* interquartile range

^*^Endometriosis, other non-colorectal malignancies including gynecological malignancies

AL was significantly influenced by procedure type. There was no AL associated with right hemicolectomies (0/111, 0%), whereas anterior resections were associated with the greatest risk of AL (high anterior resection 4/91 (4.4%), low anterior resection 5/47 (10.6%), and ultralow anterior resection 5/42 (11.9%) (*p* = 0.021). Whether the approach was performed laparoscopically or by open surgery, hand-assisted laparoscopic (hybrid) or converted to open did not influence the rate of AL in this study (*p* = 0.557). Most of the AL patients (*n* = 13/16, 81.3%) had surgery for colorectal cancer. 12.5% of these patients (*n* = 2/16) had neoadjuvant radiotherapy. None of the AL patients had pre-existing immunosuppression or hypothermia perioperatively. Other AL patient characteristics have been listed in Table 2. The indication for surgery for the majority of patients in this study was for cancer. Approximately 20% of cases were for benign indications. Cancer did not increase the risk of AL (refer to Table 1).

**Table 2:** Clinical characteristics of AL patients

	**Age (years)**	**Indication for surgery**	**Current smoker (Y/N)**	**Immuno-suppression (Y/N)**	**History of pelvic radiotherapy/neo-adjuvant radiotherapy (Y/N)**	**Other significant comorbidities**
1	66	CRC	N	N	N	-
2	71	CRC	Y	N	N	CABG, CRF
3	78	Poorly differentiated renal carcinoma invading left colon	N	N	N	Stroke, MI, CRF
4	69	CRC	N	N	N	-
5	69	CRC	Y	N	N	T2DM
6	80	Diverticular disease	N	N	N	CABG
7	66	CRC	N	N	N	T2DM
8	72	CRC	N	N	N	
9	64	CRC	N	N	N	COPD, CRF
10	69	CRC	N	N	N	-
11	43	Endometriosis	N	N	N	-
12	75	CRC	Y	N	N	T2DM
13	40	CRC	Y	N	Y	-
14	59	CRC	N	N	N	-
15	79	CRC	N	N	Y	T2DM
16	85	CRC	Y	N	N	

	**Preoperative albumin (g/L)**	**Preoperative protein (g/L)**	**Preoperative WCC (× 10**^**9**^**/L)**	**Preoperative CRP (mg/L)**	**Preoperative Hb (g/L)**	**Type of surgery**
1	34	78	5	3	109	Elective hand-assisted laparoscopic low anterior resection
2	37	-	9.5	-	133	Elective laparoscopic anterior resection
3	21	74	9.4	215	104	Semi-elective laparoscopic anterior resection
4	40	81	6.9	-	163	Elective laparoscopic anterior resection
5	-	-	9.3	-	160	Elective laparoscopic anterior resection
6	34	76	6.8	-	105	Elective laparoscopic anterior resection, repair of the bladder for colovesical fistula
7	42	81	5.9	-	124	Elective laparoscopic low anterior resection
8	37	71	5.8	4	143	Elective laparoscopic low anterior resection
9	32	77	6.1	-	75	Elective laparoscopic anterior resection
10	40	80	14.1	4	142	Emergency open high anterior resection
11	39	73	13.6	-	140	Elective laparoscopic anterior resection
12	40	69	6	-	102	Elective laparoscopic mobilization of right colon, converted to open left hemicolectomy
13	37	81	5.2	-	105	Elective laparoscopic ultralow anterior resection + loop ileostomy
14	36	66	6.4	-	133	Elective laparoscopic ultralow anterior resection
15	39	72	-	-	137	Elective laparoscopic ultralow anterior resection + loop ileostomy
16	46	86	5.2	4	154	Elective open resection of ileocolic anastomosis, segmental resection of distal transverse colon

	**Type of anastomosis**	**Duration of Surgery (mins)**	**Perioperative blood transfusions (units of blood)**	**Perioperative hypothermia (Y/N)**	**Stage of malignancy**	**Anastomotic leak (postoperative day)**
1	End to end, stapled	329	0	0	T3N0M0	12
2	End to end, stapled	182	0	0	T4aN0M0	13
3	End to end, stapled	322	4	0	Poorly differentiated renal cell carcinoma invading left colon	6
4	End to end, stapled	241	0	0	T4aN0M0	4
5	End to end, stapled	140	0	0	T3N0M0	4
6	End to end, stapled	225	1	0	Diverticular disease	9
7	End to end, stapled	278	0	0	T2N2aM0	5
8	End to end, stapled	166	0	0	T3N0M0	5
9	End to end, stapled	145	9	0	T3N0M0	20
10	End to end, stapled	245	0	0	T3N0M0	7
11	End to end, stapled	320	0	0	Endometriosis	2
12	End to end, stapled	246	0	0	T3N0M0	2
13	End to end, stapled	330	0	0	ypT3ypN1cM0	9
14	End to end, stapled	399	0	0	T3N0M0	7
15	End to end, stapled	402	0	0	ypT1ypN0M0	13
16	Side to side, stapled	183	0	0	T3N0M0	5

*CRC* colorectal cancer, *CABG* coronary artery bypass graft, *CRF* chronic renal failure, *MI* myocardial infarction, *T2DM* type 2 diabetes mellitus, *COPD* chronic obstructive pulmonary disease

**Fig. 1:**
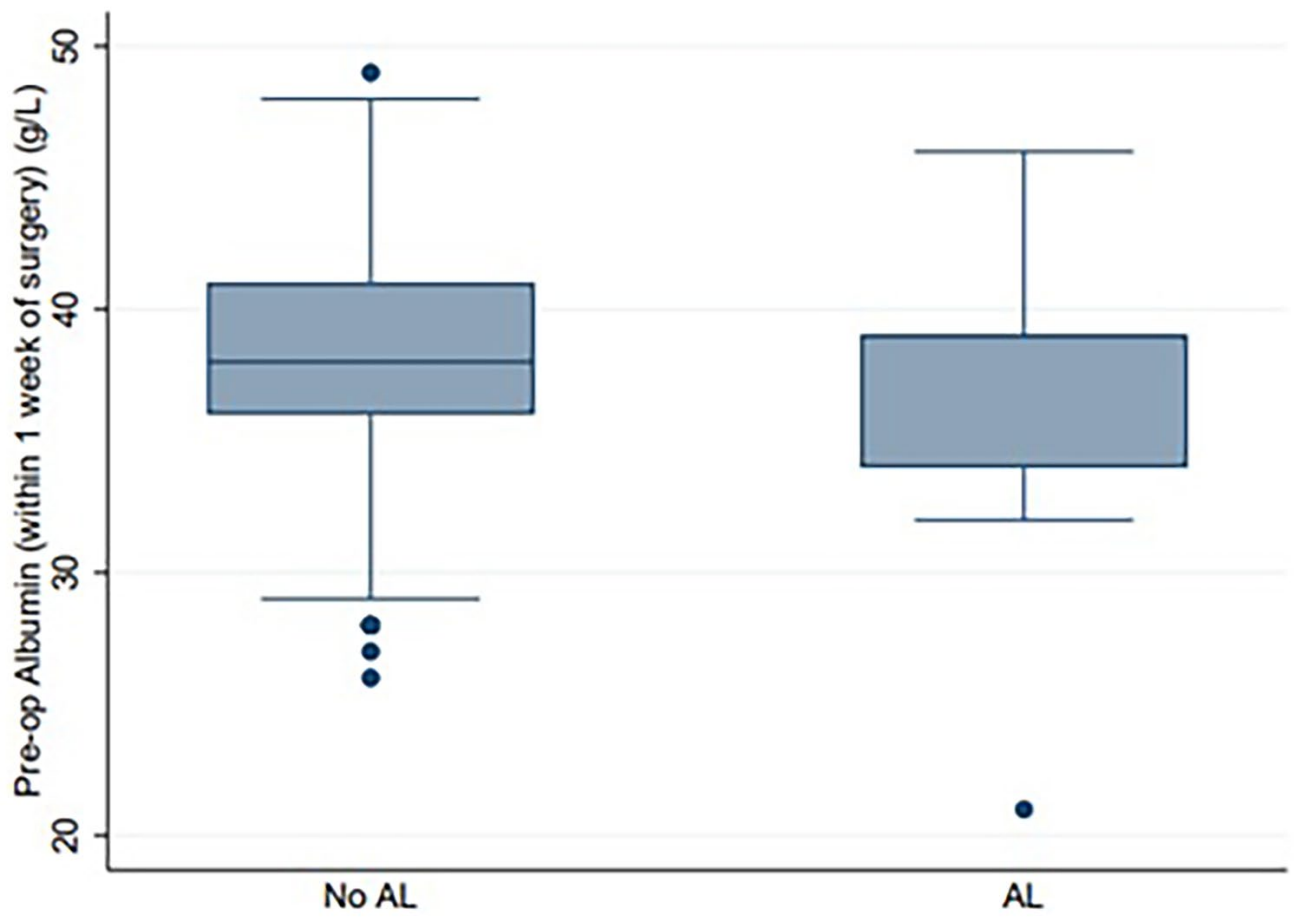
Box and Whisker plot comparing preoperative serum albumin in the no anastomotic leak vs anastomotic leak groups. Median albumin *p* = 0.4457. AL, anastomotic leak

**Fig. 2:**
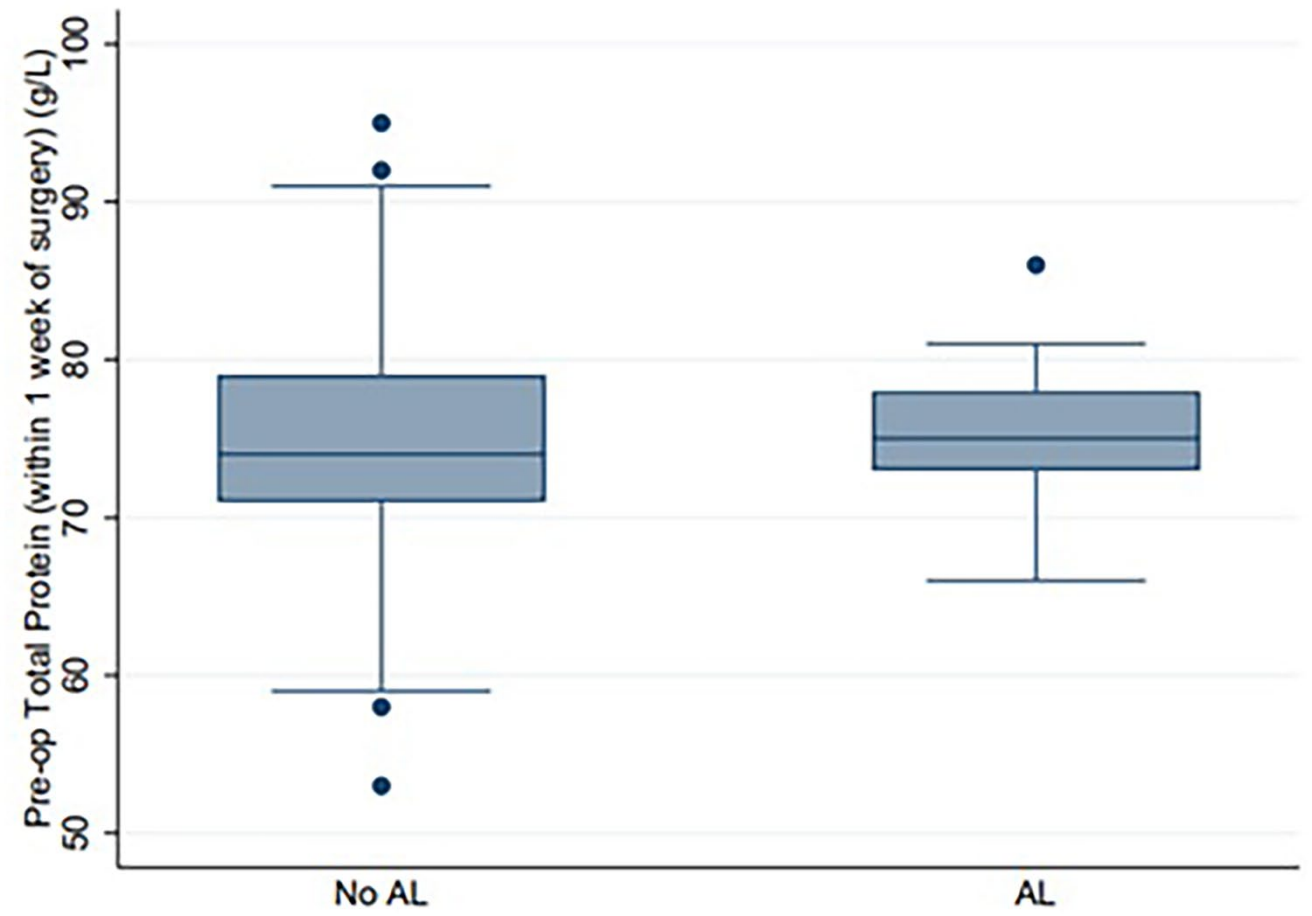
Box and Whisker plot comparing preoperative serum protein in the no AL vs AL groups. Median protein *p* = 0.6245. AL, anastomotic leak

The median preoperative albumin and protein level in the AL group were 39 g/L (normal range 35–50 g/L) and 75 g/L (normal range 60–80 g/L), respectively. The median preoperative albumin and protein level in the no AL group were 38 g/L and 74 g/L, respectively. The Mann–Whitney *U* test showed no statistically significant difference in albumin levels (*p* = 0.4457) nor protein levels (*p* = 0.6245) in the AL and no AL groups (see Table 1 and Figs. 1 and 2).

**Fig. 3:**
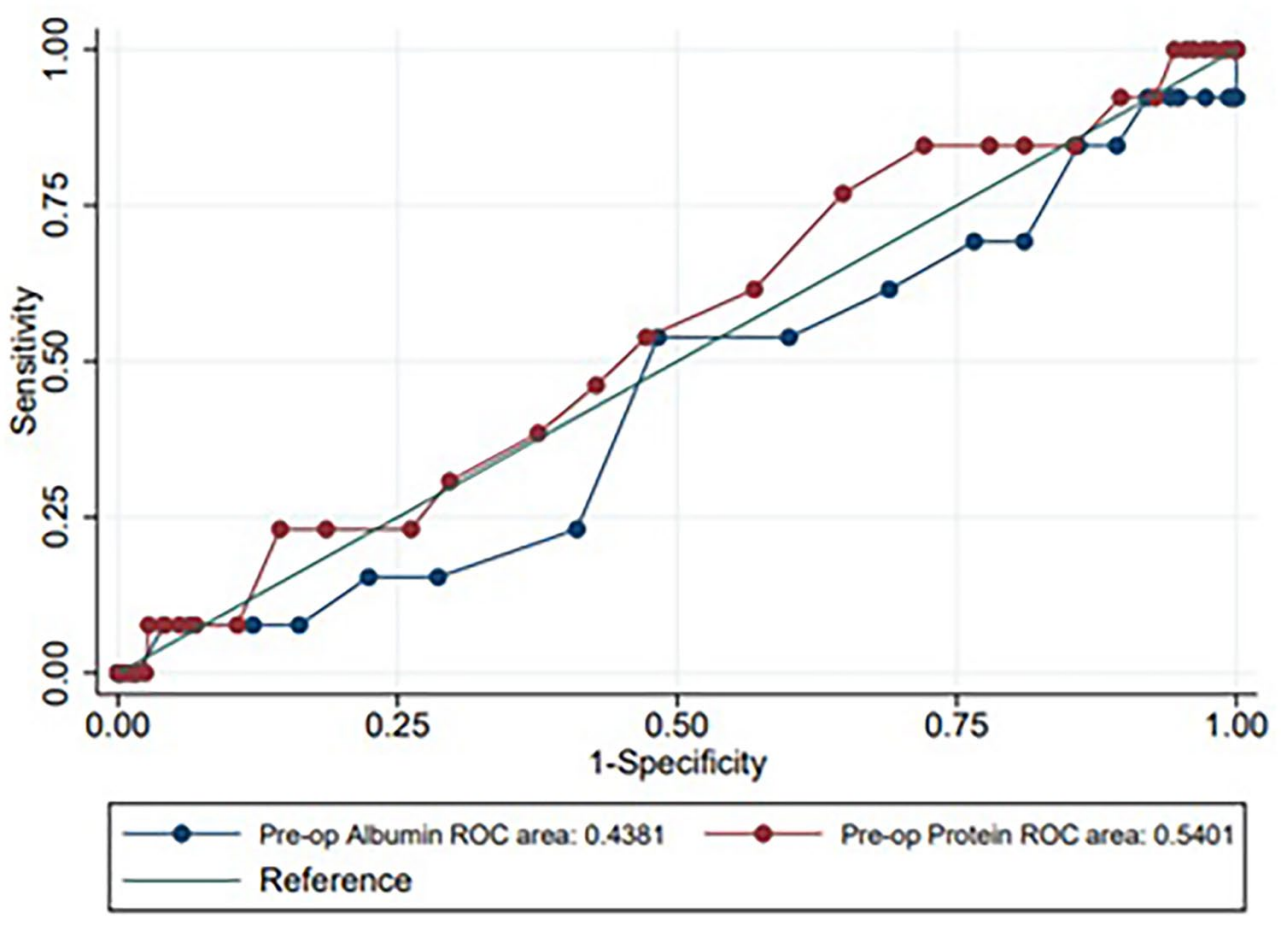
Receiver operating characteristic curve for preoperative serum albumin level (blue) and preoperative serum protein level (maroon). ROC, receiver operating characteristic

ROC curves demonstrated that preoperative albumin and protein levels were not good predictors of anastomotic leak. Cutoff values for albumin (38 g/L) and protein (75 g/L) both had poor PPV for AL (4.8% and 3.8% respectively) (see Fig. 3).

## Discussion

In this study, the AL rate was 4.4% after restorative colorectal surgery. In the surgical literature, the AL rate varies considerably, ranging from 3 to 15.9% [18, 19]. While several studies have shown the impact of nutrition on AL [20], this study did not show an association between albumin and protein level with AL. The findings of this present study provide a significant point of difference from most existing studies which have reported that pre-operative serum albumin is one of the most important predictors of AL [10, 21].

Albumin is synthesized by liver hepatocytes and rapidly excreted into the bloodstream at the rate of about 10–15 g per day. It has a half-life of approximately 20 days [22]. Its precursor prealbumin (transthyretin) is a transport protein for thyroid hormone synthesized by the liver with a half-life of 2–3 days [23]. Hypoalbuminemia is believed to affect anastomotic healing by impairing collagen synthesis due to lack of essential amino acids [24]. It is also believed to reduce host immunocompetence, increasing vulnerability for anastomotic leakage [24]. Hypoalbuminemia may manifest in both malnutrition and inflammation, by reducing its rate of synthesis [25]. In addition, inflammation alone is associated with increased synthesis of c-reactive protein from the liver and greater fractional catabolic rate of albumin, and when extreme, increases transfer of albumin out of the vascular compartment [25]. In sepsis from AL, a vicious cascade of events ensues where inflammation induces anorexia and reduces the effective use of dietary protein and energy intake, and augments further catabolism of the key somatic protein, albumin [25]. This hypothesis is supported by several studies which have shown that hypoalbuminemia is a major risk factor for AL [26–28]. In a study of 3849 colorectal cancer patients, not only was hypoalbuminemia associated with a two-fold increase in anastomotic complication rate, the 5-year overall survival was significantly reduced as well [29].

Currently, there are no established guidelines as to what level of preoperative malnutrition requires intervention. One study demonstrated that serum albumin < 35 g/L was a substantial risk factor for anastomotic leak after multivariate analysis, with an odds ratio of 13.2 (95% CI, 2.83–61.85) in the AL vs no AL groups [30]. A retrospective study of 17,518 patients concluded that a pre-operative serum albumin level < 40 g/L was associated with an increased risk of AL (*p* = 0.03) [6].

Our study is not the first study to demonstrate no difference in AL rate based on preoperative serum albumin/protein levels. A recent study of 200 colorectal cancer patients who had undergone laparoscopic surgery also demonstrated no association between preoperative serum albumin level and AL [31]. In this study, only postoperative albumin was predictive for AL—on a multivariate analysis, a lower average level of serum albumin on postoperative days 1 and 3 was predictive for AL (odds ratio 7.53, CI 1.60–55.80, p = 0.00095) [31]. Perhaps, postoperative serum albumin alongside CRP may be used to identify patients with a high index of suspicion for AL.

In the present study, the median preoperative serum albumin level was < 40 g/L in both the AL and no AL group. There was no statistical significance between the groups. Cutoff values for albumin (38 g/L) had a poor PPV for AL (4.8%).

Total serum protein levels in this study were not a predictor of anastomotic leak. This was also a point of difference from most existing studies. In the current literature, total serum protein level is believed to provide some information regarding a patient’s general health. The normal serum protein range is 60–80 g/L, of which albumin makes up 35–50 g/L, and the remainder is made up of total globulins [32].

A prospective, observational study with 1102 patients reported that a preoperative serum protein concentration of less than 60 g/L was an independent risk factor for AL after oncologic elective right colectomy [10]. Another study from the same first author performed a multicentric, prospective, national study with 3193 patients, which also found a lower median preoperative serum total protein in the AL group (65 g/L) compared to no AL group (67 g/L) in patients who underwent colectomy with primary anastomosis for colon cancer (*p* < 0.0001) [11].

It is thought that both hypoproteinemia and low hemoglobin may affect the colonic microcirculation, perfusion, and oxygenation, particularly of the anastomotic margins, and predispose to AL [20].

This present study has demonstrated that preoperative protein and albumin levels in patients undergoing elective colorectal surgery as part of an ERAS program were not reliable independent risk factors for AL. One of the major strengths of this study was that it involved a review from a prospectively collected database within a defined ERAS protocol, with very few (< 5%) missing data. A confounding factor may be related to patients receiving nutritional supplementation three times a day from 5 days prior to surgery which may help to correct nutritional deficits. Patients in our cohort routinely received “Impact” (Novartis/Nestlé), which contains 11 g of total fat, 26 g of protein, and 20 g of total carbohydrates (involving ω-3 fatty acids, arginine, and nucleotides) per 250 mls serve [33]. “Impact” is a high-protein immunonutrition drink. Nutritional supplementation preoperatively as part of an ERAS program may be protective against AL, and this may be the reason that markers of nutrition such as protein and albumin were not demonstrated to be reliable markers of AL. While compliance to “Impact” was not checked, patients were extensively counseled on the importance of preoperative nutrition, and “Impact” was provided to patients when they attended preadmission clinic as part of a quality improvement project during the time period of this study to reduce non-compliance. In any case, the median preoperative median albumin and protein levels were within the normal range for both groups (Figs. 1 and 2). Another limitation of this study was the study size was small (*n* = 361).

Systematic reviews have been published to investigate the potential benefits of immunonutrition such as “Impact” regarding postoperative outcome, with many of these reporting reductions in overall surgical complications, hospital stay, and infectious complications [34–36]. For this reason, it was added to our surgical site reduction (SSI) bundle. The rate of SSI and AL in our study was comparable to or better than similar studies in the literature [10, 26, 37].

Future studies investigating measurements of albumin, protein as well as pre-albumin in the pre-operative (both prior to nutritional supplementation and post-nutritional supplementation), and intra-operative and post-operative period may be useful to assess the impact and value of nutritional supplementation in the perioperative period in a patient undergoing major colorectal surgery.

## Conclusion

In patients undergoing elective colorectal surgery as part of an ERAS program, preoperative serum albumin and protein levels are not reliable in predicting AL. This may be because of nutritional supplementation provided as part of an ERAS program may correct nutritional deficits to protect against AL or that low albumin/protein is not as robust a marker of AL as previously reported.

## Data Availability

The authors are happy to provide data regarding this manuscript upon request.

## Declarations

A/Prof James Toh is the Chair of the ERAS Working Group, Agency for Clinical Innovation, Australia
